# Mechanisms Underlying Ectopic Persistent Tooth-Pulp Pain following Pulpal Inflammation

**DOI:** 10.1371/journal.pone.0052840

**Published:** 2013-01-16

**Authors:** Shingo Matsuura, Kohei Shimizu, Masamichi Shinoda, Kinuyo Ohara, Bunnai Ogiso, Kuniya Honda, Ayano Katagiri, Barry J. Sessle, Kentaro Urata, Koichi Iwata

**Affiliations:** 1 Department of Endodontics, Nihon University School of Dentistry, Tokyo, Japan; 2 Department of Physiology, Nihon University School of Dentistry, Tokyo, Japan; 3 Department of Oral Physiology, Faculty of Dentistry, University of Toronto, Toronto, Ontario, Canada; 4 Department of Complete Denture Prosthodontics, Nihon University School of Dentistry, Tokyo, Japan; 5 Division of Applied System Neuroscience Advanced Medical Research Center, Nihon University Graduate School of Medical Science, Tokyo, Japan; University of São Paulo, Brazil

## Abstract

In order to clarify the peripheral mechanisms of ectopic persistent pain in a tooth pulp following pulpal inflammation of an adjacent tooth, masseter muscle activity, phosphorylated extracellular signal-regulated protein kinase (pERK) and TRPV1 immunohistochemistries and satellite cell activation using glial fibrillary acidic protein (GFAP) immunohistochemistry in the trigeminal ganglion (TG) were studied in the rats with molar tooth-pulp inflammation. And, Fluorogold (FG) and DiI were also used in a neuronal tracing study to analyze if some TG neurons innervate more than one tooth pulp. Complete Freund’s adjuvant (CFA) or saline was applied into the upper first molar tooth pulp (M1) in pentobarbital-anesthetized rats, and capsaicin was applied into the upper second molar tooth pulp (M2) on day 3 after the CFA or saline application. Mean EMG activity elicited in the masseter muscle by capsaicin application to M2 was significantly larger in M1 CFA-applied rats compared with M1 vehicle-applied rats. The mean number of pERK-immunoreactive (IR) TG cells was significantly larger in M1 CFA-applied rats compared with M1 vehicle-applied rats. Application of the satellite cell inhibitor fluorocitrate (FC) into TG caused a significant depression of capsaicin-induced masseter muscle activity and a significant reduction of satellite cell activation. The number of TRPV1-IR TG cells innervating M2 was significantly larger in M1 CFA-applied rats compared with M1 vehicle-applied rats, and that was decreased following FC injection into TG. Furthermore, 6% of TG neurons innervating M1 and/or M2 innervated both M1 and M2. These findings suggest that satellite cell activation following tooth pulp inflammation and innervation of multiple tooth pulps by single TG neurons may be involved in the enhancement of the activity of TG neurons innervating adjacent non-inflamed teeth that also show enhancement of TRPV1 expression in TG neurons, resulting in the ectopic persistent tooth-pulp pain following pulpal inflammation of adjacent teeth.

## Introduction

It is well known that ectopic persistent pain is frequently observed in teeth or other intraoral structures following tooth-pulp inflammation [1]. The presence of persistent pain in a tooth associated with pulpal inflammation in an adjacent tooth sometimes accounts for misdiagnosis of the source of the persistent pain and inappropriate treatment of non-inflamed healthy teeth. For the appropriate treatment of the inflamed tooth pulp, it is necessary to diagnose the teeth under pathological conditions. Although neuroplastic changes in the central nervous system (CNS) and/or sensitization of afferents in the peripheral nervous system (PNS) are thought to be involved in the ectopic pulpal pain following tooth-pulp inflammation [2], [3], the peripheral mechanisms underlying ectopic pulpal pain following tooth-pulp inflammation are not fully understood.

The CNS mechanisms underlying ectopic pain following peripheral inflammation have been reported in many studies [4], [5], [6], [7]. Following peripheral inflammation, a barrage of action potentials are generated in primary afferent neurons which are sent to the CNS, resulting in the sensitization of nociceptive neurons in the spinal dorsal horn (SDH) as well as primary afferent neurons [8], [9], [10]. A number of previous studies have reported that the enhancement of spontaneous activity, lowered threshold to evoke firings and expansion of the receptive field are evident in SDH nociceptive neurons following leg muscle or knee joint inflammation [8], [11], [12]. The hyperexcitability of SDH neurons are thought to be involved in neuroplastic changes in the SDH neuronal circuitry following peripheral inflammation that are associated with ectopic persistent pain in the adjacent non-inflamed tissues.

On the other hand, some recent papers have reported that trigeminal ganglion (TG) neurons have functional interactions each other, and neuronal interactions among TG neurons are thought to be involved the enhancement of noxious responses in primary afferent neurons innervating in non-inflamed orofacial tissues following inflammation or injury of adjacent tissues [13], [14]. Following temporomandibular joint (TMJ) inflammation, substance P (SP) production is accelerated and released from the small-diameter primary afferent neurons in TG, and affects the excitability of intact TG neurons [13]. Furthermore, nerve growth factor (NGF) is also released from TG neurons innervating the inflamed tissues and TRPV1 production is enhanced in TG neurons, and may affect TG neuronal activity innervating non-inflamed upper lip following complete Freund’s adjuvant (CFA) application to other parts of the lip [4]. The enhancement of TRPV1 upregulation has also been reported in TG neurons following tooth pulp inflammation [15]. These findings indicate that various molecules are released from TG neurons following orofacial inflammation, and TRPV1 production was accelerated in TG neurons innervating non-inflamed tissues, resulting in the enhancement of TG neuronal excitability. It has also been reported that satellite cell activation is significantly enhanced in the wide area of TG following lingual nerve crush [16]. These activated satellite cells are also known to release various molecules such as SP, calcitonin gene-related peptide (CGRP) or brain-derived neurotrophic factor (BDNF) [17], and there is strong evidence that neuron-glia interactions in TG are involved in the enhancement of neuronal excitability of intact TG neurons following orofacial inflammation.

Extra cellular-signal regulated kinase (ERK) is known to be phosphorylated in dorsal root ganglion (DRG) neurons via Ca^2+^ influx following strong noxious stimulation of the hind paw, and the number of phosphorylated ERK-immunoreactive (pERK-IR) cells is increased following increases in the noxious stimulus intensity [18], suggesting that ERK phosphorylation could be used as the marker of the excitability of TG neurons following noxious stimulation of the peripheral tissues. Furthermore, it is highly possible that TRPV1 production is accelerated in TG neurons following pulpal inflammation and is involved in ectopic tooth-pulp pain based on our previous study [4]. Thus, we applied ERK phosphorylation as a maker of excitability of TG neurons following capsaicin application to the upper second molar tooth pulp (M2) in the rats with CFA- or vehicle-application to the upper first molar tooth pulp (M1).

To clarify mechanisms underlying ectopic persistent pulpal pain following tooth-pulp inflammation, we analyzed the masseter muscle activity to evaluate noxious reflex, pERK immunohistochemistry to detect excitable TG neurons, glial fibrillary acid protein (GFAP) immunohistochemistry to study satellite cell activation and primary afferent tracings using fluorogold (FG) and DiI applied respectively into M1 or M2 to study the percentage of single TG neurons supplying more than one tooth pulp, and we also studied TRPV1 expression TG neurons in rats with CFA-induced tooth-pulp inflammation.

## Materials and Methods

This study was approved by the Animal Experimentation Committee at Nihon University. All surgery and animal care were conducted in accordance with the National Institutes of Health Guide for the Care and Use of Laboratory Animals and the guidelines for Institutional Animal Care, and the guidelines of the International Association for the Study of Pain [19].

Male Sprague-Dawley rats (n = 177, Japan SLC, Shizuoka, Japan) weighing 250–350 g were used in this study. The animals were maintained in a temperature-controlled room (23°C) with a 12/12 hour light-dark cycle. Food and water were freely available.

### Tooth Pulp Inflammation Model

Rats were lightly anesthetized with 2% isoflurane (Mylan, Canonsburg, PA) and then deeply anesthetized with an intraperitoneal (i.p.) application of sodium pentobarbital (50 mg/kg; Schering Plough, Whitehouse Station, NJ). Then the rats were placed on a warm mat (37°C) in the dorsal recumbent position to allow for the application of CFA (Sigma-Aldrich, St. Louis, MI). Fifty % CFA (dissolved in saline) or vehicle (isotonic physiological saline) was applied to M1 unilaterally. The rats' mouth was gently opened and the dental pulp was exposed by means of a low-speed dental drill with a round tungsten carbide bur under water cooling. A small piece of dental paper point (diameter, 0.15 mm; length, 1.5 mm) soaked with CFA or saline was applied to the exposed tooth pulp. Then the exposed pulp cavity was sealed with dental cement.

To evaluate if the inflammation occurs in the periodontal tissue around the apex 3 days after CFA application to M1, we studied CFA-applied M1 sections. Three days after CFA application to M1, rats were anesthetized with sodium pentobarbital (50 mg/kg, i.p.) and perfused with 4% paraformaldehyde. Maxillar bone and M1 were removed and decalcified overnight and 20 µm serial sections were cut. Then sections were stained with hematoxylin and eosin and coverslipped.

### EMG Recording After Capsaicin Application to M2

On day 3 after the CFA or saline administration to M1, rats were anesthetized with 3% isoflurane and M2 was exposed for capsaicin administration, and a pair of bipolar wire electrodes (enamel-coated stainless steel wire, inter-polar distance: 5 mm) was inserted into the masseter muscle on the side ipsilateral to the M2 tooth pulp exposure. After that, the M2 pulp surface was covered with a small piece of cotton soaked with isotonic physiological saline until capsaicin was applied. After surgery, the isoflurane concentration was reduced (0.6–0.8%) and this anesthetic level was maintained during experiment. The heart beat rate and body core temperature were continuously monitored throughout the experiment and kept within the physiological range.

Masseter muscle EMG activity elicited by capsaicin application to the tooth pulp was analyzed according to the procedure reported by Sunakawa et al [20]. A rest period of 30 min was allowed after the surgery in order to obtain a stable baseline level of EMG activity. EMG activity in jaw muscle was continuously monitored before, during and after the application of capsaicin into M2. EMG activity was monitored for 20 min before application of capsaicin (i.e. baseline) and for an additional 20 min after capsaicin application. The EMG activity from masseter muscle was amplified, rectified and integrated, and the area under the curve of EMG activity was calculated by the Spike 2 software (CED, Cambridge,UK). The area under the EMG activity was measured for every 1 min before and after capsaicin application, and mean EMG activity was calculated for each time period. The mean baseline level of EMG activity was determined from the mean EMG activity 3 min prior to the application of capsaicin to M2. We also studied the effect of PD98059 as the MEK1 inhibitor to test its effect on masseter muscle EMG activity elicited by capsaicin application to the tooth pulp.

Capsaicin was applied to M2 unilaterally by a small piece of dental paper point soaked with capsaicin (Wako, Osaka, Japan). Capsaicin (3.125 mg) was diluted with Tween 80 (65.6µl), 100% ethanol (62.5µl) and saline (871.8µl) (10 mM).

### pERK, NeuN,GFAP and TRPV1 Immunohistochemistries

On day 3 after the CFA or saline application to M1, rats were lightly anesthetized with 2% isoflurane and then deeply anesthetized with sodium pentobarbital (50 mg/kg, i.p.), and the M2 tooth pulp was exposed. After a rest period of 30 min, capsaicin was applied to M2, and 5 min later the rats were transcardially perfused with isotonic saline followed by a fixative containing 4% paraformaldehyde in 0.1 M phosphate buffer (PB, pH 7.4), and the ipsilateral TG was removed and was processed for pERK or Neuronal Nuclei (NeuN) immunohistochemistry. The same amount of solvent (100% ethanol and 7% Tween 80 in saline) was used as a vehicle.

TGs ipsilateral side to the M1 operation were dissected out after perfusion and immersed in the same fixative for 1day at 4°C, and then kept in 0.01 M phosphate-buffered saline (PBS) containing 20% sucrose (w/v) for 12h for cryoprotection. The specimens were then embedded in Tissue Tek (Sakura Finetek, Torrance, CA) and stored until cryosectioning at −20°C. Ten µm horizontal plane sections were cut from TG, and sections were thaw-mounted on MAS-GP micro slide glass (Matunami, Osaka, Japan) and dried overnight at room temperature.

For GFAP and TRPV1 immunohistochemistries, on day 3 after CFA or saline application to M1, rats were lightly anesthetized with 2% isoflurane and then deeply anesthetized with sodium pentobarbital (50 mg/kg, i.p.), and rats were transcardially perfused with the same fixative used for pERK and NeuN immunohistochemistries.

For pERK immunohistochemistry, sections were incubated in 10% normal goat serum (NGS) in PBS for 1 h, and then incubated in rabbit antiphospho-p44/42 MAP Kinase Antibody (1∶200, Cell Signaling Technology, Danvers, MA) for 72 h at room temperature. Next, the sections were incubated in biotinylated goat anti-rabbit IgG (1∶600; Vector Labs, Burlingame, CA) for 2 h at room temperature. After washing, the sections were incubated in peroxidase-conjugated avidin–biotin complex (1∶100; Vector Labs) for 2 h at room temperature. After washing in 0.05 M Tris Buffer (TB), the sections were incubated in 0.035% 3,3-diaminobenzidine-tetra HCl (DAB, Sigma-Aldrich), 0.2% nickel ammonium sulfate and 0.05% peroxide in 0.05 M TB (pH 7.4). The sections were washed in 0.01 M PBS, serially mounted on gelatin-coated slides, dehydrated in alcohols and coverslipped. We also studied ERK phosphorylation in TG neurons following CFA or saline application to M1. Furthermore, the effect of vehicle or capsaicin application to M2 was studied in naïve rats.

For double immunofluorescence histochemistry for pERK and NeuN, sections were rinsed in PBS, 10% normal goat serum in PBS for 1 h, and then incubated in rabbit antiphospho-p44/42 MAP Kinase Antibody (1∶200, Cell Signaling Technology) for 72 h at room temperature. Then the sections were incubated in mouse anti-neuronal nuclei monoclonal Antibody (1∶500, Chemicon, Temicula, CA) for 2h at room temperature. Next, the sections were incubated in Alexa Fluor 488 anti-rabbit IgG (1∶200 in 0.01 M PBS; Invitrogen, Paisley, UK) and in Alexa Fluor 568 anti-mouse IgG (1∶200 in 0.01 M PBS; Invitrogen) for 2h at room temperature. After rinsing with 0.01 M PBS, sections were coverslipped in mounting medium (Thermo Fisher Scientific, Fremont, CA) and examined under a fluorescence microscope and analyzed using a BZ-9000 system (Keyence, Osaka, Japan).

For GFAP immunohistochemistry, sections were incubated with mouse anti-GFAP monoclonal antibody (Chemicon) after dilution at a concentration of 1∶500 in 0.01 M PBS containing 4% NGS and 0.3% Triton X-100 (Sigma-Aldrich) overnight at 4°C. After rinsing with 0.01 M PBS, sections were incubated in Alexa Fluor 488 anti-mouse IgG (1∶200 in 0.01 M PBS; Invitrogen) for 2h at room temperature. The number of somata of TG neurons encircled with GFAP-IR cells over 2/3 of the soma perimeters of neurons (SensivMeasure; Mitani, Fukui, Japan) was counted in each rat (n = 5 in each group) and the relative number of them was calculated by the following formula: 100× number of neurons encircled with GFAP-IR cells/total number of neurons.

TRPV1 immunohistochemical analyses were conducted in vehicle- and CFA-treated rats to locate TRPV1 in TG (n = 5 in each group). Sections were incubated with rabbit anti-TRPV1 polyclonal antibody (1∶500; Alomone labs, Jerusalem, Israel), in 0.01 M PBS containing 4% NGS and 0.3% Triton X-100 (Sigma-Aldrich) overnight at 4°C. After rinsing with 0.01M PBS, sections were incubated in Alexa Fluor 488 anti-rabbit IgG (1∶200 in 0.01M PBS; Invitrogen) for 2h at room temperature. After rinsing with 0.01M PBS, sections were coverslipped in mounting medium and examined under a fluorescence microscope. We also analyzed the effect of FC injection into the TG on TRPV1-IR cell expression in M1 CFA-applied rats.

### pERK, GFAP and TRPV1 Immunohistochemistries in Combination with FG Tracer

For pERK immunohistochemistry in combination with FG tracer into M1, on day 3 after CFA or saline mixed with FG application to M1, rats were anesthetized, and 5 min after capsaicin application to M2, they were perfused with the same fixative as above. TG sections were processed with pERK fluorescent immunohistochemistry as described above.

For GFAP immunohistochemistry in combination with FG traces into M1, on day 3 after CFA or saline mixed with FG application to M1, rats were anesthetized, and perfused with same fixative and TG sections were processed with GFAP fluorescent immunohistochemistry, and FG-labeled (+) GFAP-IR cells and FG-unlabeled (–) GFAP-IR cells were analyzed.

For TRPV1 immunohistochemistry in combination with FG trace into M2, rats received FG application to M2 and CFA or saline into M1 under deep anesthesia, on day 3 after M1 and M2 applications rats were deeply anesthetized, and perfused with same fixative. Then, TG sections were processed withTRPV1 fluorescent immunohistochemistry, and FG(+) TRPV1-IR cells were also examined. We also examined immunohistochemical staining in each section without primary antibodies, and no immunoreactive cells were observed in all sections.

To evaluate if FG spread to the periodontal tissue through the apex of M1, we studied FG-applied M1 sections histologically. Three days after FG application to M1, rats were anesthetized with sodium pentobarbital (50 mg/kg, i.p.) and perfused with 4% paraformaldehyde. Maxillar bone and M1 were removed and decalcified overnight and 20 µm serial sections were cut and coverslipped.

### FG and DiI Tracings

Rats were deeply anesthetized, and 10% of FG (Wako) dissolved in saline was applied into M1 and DiI (Invitrogen) saturated in 100% ethanol was also applied into M2. Subsequently on day 3, rats were deeply anesthetized and TG was removed and sectioned. FG- and/or DiI-labeled cells were studied in TG under fluorescent microscopy.

### PD98059 or Fluorocitrate Administration into TG

Rats were anesthetized with 2% isoflurane in oxygen and sodium pentobarbital (50 mg/kg, i.p.) and placed in a stereotaxic apparatus. The skull was exposed and a small hole (diameter; 1 mm) was drilled above the location of the bifurcation between 1^st^ and 2^nd^ branches of the trigeminal nerve (V1/V2) branch regions and 3^rd^ branch of the trigeminal nerve (V3) branch region of TG. The guide cannula was extended into the hole 9 mm below the skull surface into TG ipsilateral to the operation (2.8 mm anterior from lambda and 2.7 mm lateral to the midline) and was fixed to the skull with three stainless-steel screws and dental cement. The tip of the trocar was located just below the surface of V1/V2 branch region near the border between V3 and V1/V2 branches to inject drugs into TG. To define the position of the tip of the cannula, multiunit activities by mechanical stimulation of the V1/V2 face area were recorded by using the trocar as an electrode. After completion of the surgery, penicillin G potassium (20,000 units; Meiji Seika, Tokyo, Japan) was injected intramuscularly to prevent infection. The rats were allowed to recover for 7 days before experiments were performed [21], [22].

Rats were lightly anesthetized with 2% isoflurane in oxygen, a 31-gauge injection needle (Heraeus Kulzer Japan, Osaka, Japan) was inserted into the TG through the guide cannula after the trocar was removed. The injection needle was connected to the 10 µl Hamilton syringe to deliver 0.5 µl drugs over 10 min. Rats were administered 100 fmol of the Fluorocitrate (FC, Sigma-Aldrich) [23] or vehicle (PBS) (n = 6 in each group) for 30 min before the EMG or immunohistochemical experiment.

PD98059 was dissolved in 10% DMSO and saline solution (0.1 µg/µl), and was administrated into TG through the guide cannula which was extended into the hole 9 mm below the skull surface into TG. Three days after CFA application into M1, PD98059 was injected into TG for 3 days (0.5µl each day) and then capsaicin was applied to the M2, and EMG activity was recorded. To study the effect of FC on TRPV1 expression in TG, 3 days after CFA application into M1, FC was injected into TG for 3 days (0.5µl each day) and perfused for TRPV1 immunohistochemistry.

### Statistical Analysis

Data were expressed as mean ± SEM. Statistical analyses were performed by Student’s *t*-test, one-way analysis of variance (ANOVA) followed by Bonferroni or Dunnet test. We also used two-way repeated-measures ANOVA followed by Fisher LSD where appropriate. A value of *p*<0.05 was considered as significant.

## Results

### 1. Masseter Muscle Activity Elicited by Capsaicin Application to M2

**Figure 1:**
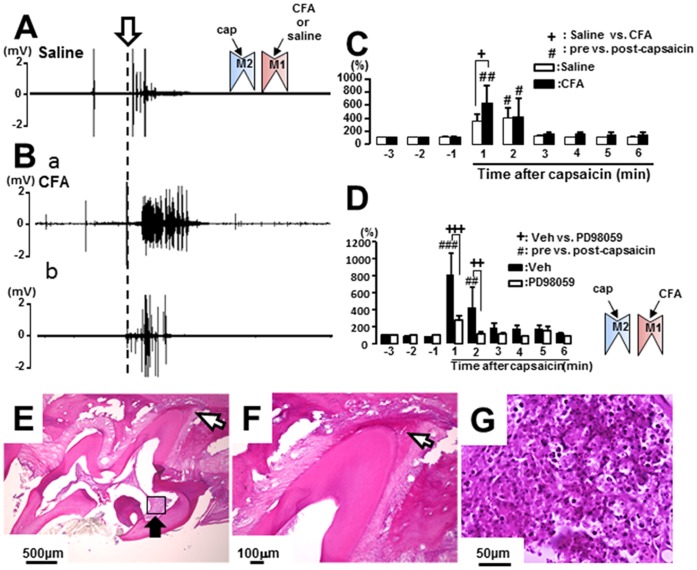
Masseter muscle activity following capsaicin application to M2 in the rats with M1 CFA-applied or saline-applied rats. Typical examples of EMG activities recorded from the masseter muscle following capsaicin application to M2 in the rats with saline (A) or CFA (Ba: large response, Bb: small response) application to M1 on day 3 after the treatments. C and D: The mean area under the curve of integrated EMG following capsaicin application to M2 in M1 CFA- or saline-applied rats (C), and that following Veh- or PD98059-administration in the rats with M1 CFA application (D). E and F: Low (E) and high (F) magnification photomicrographs of CFA-applied M1 sections. G: High magnification photomicrograph of the area inidicated by the square box with the solid arrow in E. The open arrow in A indicates the timing of capsaicin application to M2. Open arrows in D and E indicate the apex of M1. The inset diagrams in A and D indicate the tooth pulp with drug application in this and following figures. CFA: complete Freund’s adjuvant, pre: before capsaicin application, post-capsaicin: after capsaicin application. +, # : p<0.05, ##: p<0.01.

The masseter muscle EMG activity after capsaicin application to M2 was measured in lightly anesthetized rats (0.6–0.8% isoflurane with oxygen). An EMG burst was observed following capsaicin application to M2 in the rats with CFA or vehicle application to M1 (Fig. 1A). Following CFA application to M1, a large EMG burst was elicited by capsaicin application to M2 (Fig. 1B). The mean area under the curve of the integrated EMG bursts evoked by capsaicin application to M2 was significantly larger in M1 CFA-applied and vehicle-applied rats compared with those before capsaicin application (n = 7 in each group) (Fig. 1C). Furthermore, the EMG activity was significantly suppressed following microinjection of PD98059 into the TG in M2 capsaicin-applied rats (n = 5 in each group) (Fig. 1D).

We observed obvious inflammation-related blood cells in the coronal pulpal tissue and could not observe inflammation in the periodontal tissue around the apex of the tooth 3 days after CFA application to M1, indicating that periodontal tissue was not inflamed (Fig. 1E, F and G).

### 2. ERK Phosphorylation in TG Neurons

**Figure 2:**
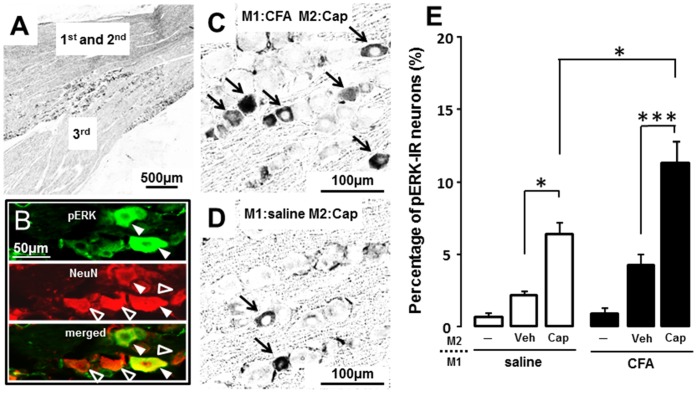
pERK-IR cells in TG following capsaicin application to M2 in the rats with CFA or saline-application to M1. A: Low magnification photomicrograph of TG in M1 CFA-applied rats. B: pERK- and NeuN-IR cells in TG following capsaicin application to M2 in the rats with CFA application to M1. C: High magnification photomicrograph of pERK-IR cells in M1 CFA-applied rats. D: High magnification photomicrograph of pERK-IR cells in M1 saline-applied rats. E: The mean percentage of pERK-IR cells in TG of M2 untreated/M1 saline, M2 Veh/M1 saline, M2 Cap/M1 saline, M2 untreated/M1 CFA, M2 Veh/M1 CFA or M2 Cap/M1 CFA rats. White arrow heads indicate pERK- and NeuN-IR cells in B. Open arrow heads indicate NeuN-IR cells in B. The solid arrow heads indicate pERK-IR cells in C and D. Veh: vehicle for capsaicin, Cap: capsaicin. * : p<0.05, ***: p<0.001.

ERK phosphorylation occurred in small TG neurons in V1/V2 blanch region after capsaicin application to M2 in M1 CFA-applied (Fig. 2A and B) or M1 vehicle-applied rats (Fig. 2D). All pERK-IR cells showed NeuN-IR, indicating that pERK-IR cells were neurons (Fig. 2B). We observed ERK phosphorylation in the small number of TG neurons following CFA or vehicle application to the M1 tooth pulp, and the number of pERK-IR cells was slightly larger in M1 CFA-applied rats than vehicle-applied rats (M1 saline/M2 untreated: 0.6±0.3, M1 CFA/M2 untreated: 0.9±0.4, n = 5 in each group) (Fig. 2E). A large number of pERK-IR cells was observed in TG following capsaicin application to M2 (M1 saline/M2 capsaicin: 6.4±0.8, M1 CFA/M2 capsaicin: 11.3±2.0, n = 7 in each group) (Fig. 2 E). On the other hand, ERK phosphorylation occurred in few TG neurons following vehicle application to M2 (M1 saline/M2 vehicle: 2.1±0.3, M1 CFA/M2 vehicle: 4.2±0.8, n = 7 in each group) (Fig. 2 E). Furthermore, significantly larger number of pERK-IR cells was observed in M2 capsaicin applied rats (5.8±0.4) than M2 vehicle applied rats (1.8±0.4) (p<0.01, n = 5 in each group). The mean relative number of pERK-IR cells was significantly larger in the rats with M2 capsaicin-application compared with M2 Veh (vehicle for capsaicin) rats in M1 CFA- or M1 vehicle-applied rats (Fig. 2E). Following CFA application to the M1 tooth pulp, the mean relative number of pERK-IR cells following capsaicin application to M2 was significantly larger in M1 CFA-applied rats compared with M1 vehicle-applied rats (Fig. 2E).

**Figure 3:**
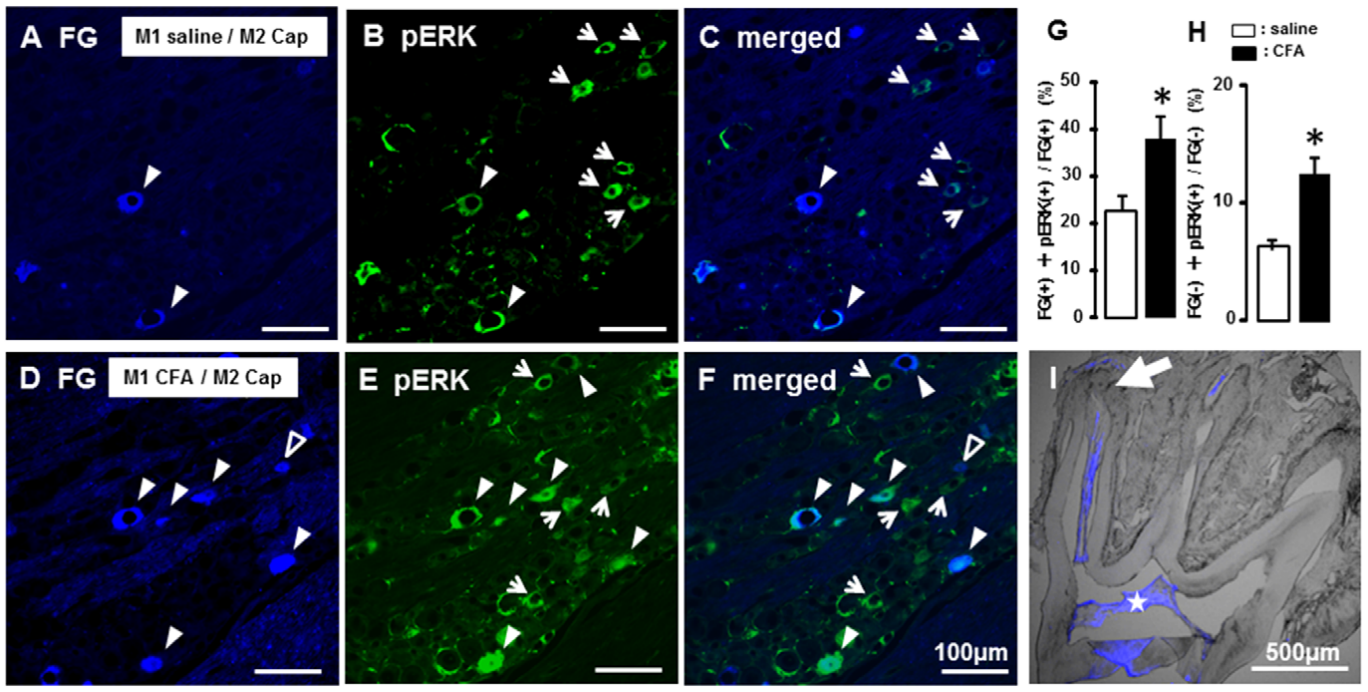
FG-labeled TG cells and pERK-IR TG cells following capsaicin application to M2 in M1 CFA- or saline-applied rats. Fluorescent photomicrographs of FG(+) cells (A and D), pERK-IR cells (B and E) and FG merged with pERK-IR (C and F) in TG (M1 saline-applied rats: A, B and C; M1CFA-inected rats: D, E and F ). G and H: The mean percentages of FG (+) pERK-IR cells/FG (+) cells (G) and mean percentage of FG(–) pERK-IR cells/FG (–) cells (H). I: The photomicrograph of FG-applied M1 sections. FG can be seen as blue-stained product in M1. The solid arrow heads indicate FG(+) pERK-IR cells in A-F. The open arrow heads indicate FG(+) cells in D and F. The arrows indicate pERK-IR cells in B, C, E and F. The large white arrow in I indicates the apex of M1. The white star in I indicates coronal pulp cavity. * : p<0.05.

To study if any pERK-IR TG cells following capsaicin application to M2 were innervated by M1 into which CFA had been applied, FG mixed with CFA or saline was applied into M1. Following capsaicin application to M2, there were many TG neurons showing pERK-IR in M1 CFA- or saline-applied rats (Fig. 3B and E), and some of them were also labeled retrogradely with FG applied into M1 (Fig. 3A-F). The mean relative number of FG(+) and FG(–) pERK-IR cells was significantly larger in M1 CFA-applied rats compared with M1 vehicle-applied rats (FG(+), saline: 22.8±3.2, CFA: 38.0±5.0; FG(–), saline: 6.3+0.7, CFA: 12.6±1.5, n = 7 in each group) (Fig. 3G and H). We did not observe FG spreading to the periodontal tissue through the apex of M1 on day 3 after FG application, indicating that FG was restricted in the M1 tooth pulp (Fig. 3I).

### 3. Satellite Cell Activation Following Tooth-pulp Inflammation

**Figure 4:**
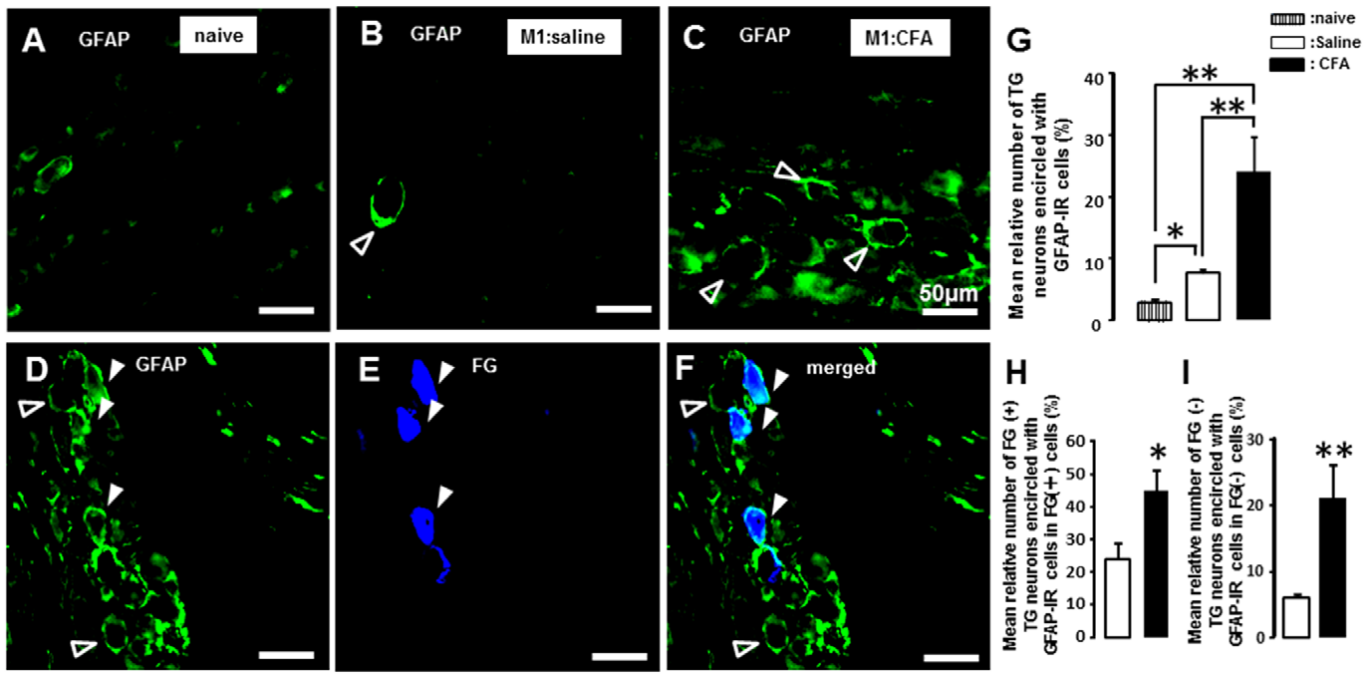
GFAP-IR cells in TG following capsaicin application to M2 in M1 CFA- or saline-applied rats. A, B and C: GFAP-IR cells in naïve (A), M1 saline (B) or CFA-applied (C) rats. D: Photomicrograph of GFAP-IR cells (D), FG(+) cells (E) and FG(+) GFAP-IR cells (F) in TG. G: The mean relative number of TG neurons encircled with GFAP-IR cells (%). H: The mean relative number of FG(+) TG neurons encircled with GFAP-IR cells in FG(+) cells (%). I: The mean relative number of FG(–) TG neurons encircled with GFAP-IR cells in FG(–) cells (%). The open arrow heads indicate GFAP-IR cells. The white arrow heads indicate FG(+) cells encircled with GFAP-IR cells. *: p<0.05, **: p<0.01.

We also studied if GFAP-IR cells were present in TG in rats with CFA applied into M1. GFAP-IR cells were observed as cells encircling TG neurons (Fig. 4A, B and C), and many of FG(+) cells were encircled with GFAP-IR cells in the TG (Fig. 4D, E and F). The mean relative number of TG cells encircled with GFAP-IR cells was significantly larger in M1 CFA-applied rats compared with M1 saline-applied rats or naïve rats (CFA: 23.7±4.5, n = 7, saline: 7.4±0.8, n = 7, naïve: 2.9±0.4, n = 5), and that was also significantly larger in saline-applied rats compared with naïve rats (Fig. 4G). The number of TG cells encircled with GFAP-IR cells was also significantly larger in M1 saline-applied rats compared with naïve rats. The mean relative number of both FG(+) and FG(–) TG cells encircled with GFAP-IR cells was significantly larger in M1 CFA-applied rats compared with M1 saline-applied rats (FG(+): saline 23.8±5.4, CFA: 44.8+6.2; FG(–): saline: 5.9+0.7, CFA: 21.0+5.2, n = 5 in each group) (Fig. 4H and I).

### 4. Effect of FC on Masseter Muscle Activity and GFAP-IR Cell Expression in TG

**Figure 5:**
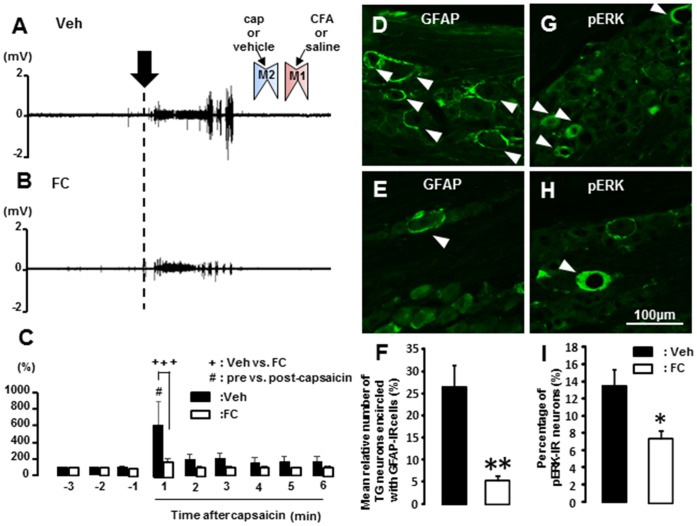
Effect of FC injection into TG on masseter muscle activity and GFAP and pERK-IR cell expression. A and B: Typical example of masseter muscle EMG activities following capsaicin application to M2 in M1 CFA-applied rats with Veh (A) or FC (B) injection to TG. C: The mean area under the curve of integrated EMG following capsaicin application to M2 in M1 CFA-applied rats with Veh or FC-injection to TG (C). D and E: Photomicrographs of GFAP-IR cells in TG Veh-injected rats (D) and TG FC-injected rats (E). F: The mean relative number (%) of TG cells encircled with GFAP-IR cells following Veh- (solid bur) or FC- (open bur) administration in M1 CFA rats. G and H: Photomicrographs of pERK-IR cells following M2 capsaicin application in TG Veh- (G) and TG FC-injected rats (H). I: The mean relative number (%) of pERK-IR cells in TG following M2 capsaicin application in TG Veh- and TG FC-injected rats which received CFA in M1. Solid arrow in A indicates the timing of capsaicin application. White arrow heads in D and E indicate GFAP-IR cells and those in G and H are pERK-IR cells, respectively. Veh: vehicle for FC, FC: Fluorocitrate. #: p<0.05, +++: p<0.001, *: p<0.05, **: p<0.01.

There was a large EMG burst induced by capsaicin application to M2 in rats with saline application to TG (Fig. 5A), but EMG burst evoked by capsaicin application to M1 was smaller in the rats with FC injection to TG (Fig. 5B). The mean area under the EMG curve at 1 min after capsaicin application to M2 was significantly smaller in FC-injected rats compared with vehicle-injected rats (n = 5 in each group) (Fig. 5C). Many TG cells encircled with GFAP-IR cells (Fig. 5 D) and pERK-IR cells following M2 capsaicin application (Fig. 5 G) were observed in rats with vehicle injection into TG following CFA application to M1. On the other hand, only a small number of TG cells encircled with GFAP-IR cells and pERK-IR cells was observed in FC-injected rats with M1 CFA-application (Fig. 5E and H). The mean number of TG cells encircled with GFAP-IR cells was significantly smaller in FC-injected M1-inflamed rats compared with vehicle-injected M1-CFA rats (vehicle: 26.6±4.8, FC: 5.4±1.0, n = 5 in each group) (Fig. 5F). There was also a significant reduction of the number of pERK-IR TG cells in rats with FC injection compared with those with vehicle application to the TG (vehicle: 13.5±1.9, FC: 7.4±0.9, n = 5 in each group) (Fig. 5 I).

### 5. FG and DiI Tracing in TG neurons

**Figure 6:**
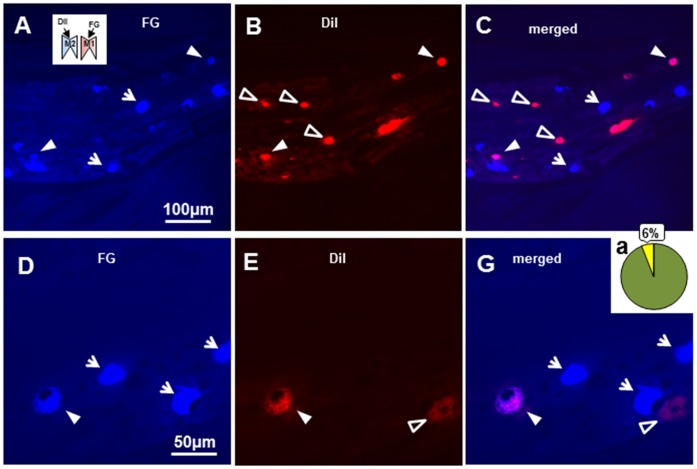
Photomicrographs and percentage of double retrograde tracing from M1 and M2 afferents to TG neurons. A, B and C: Low magnification photomicrographs of FG(+) TG cells at 3 days after FG application to M1 (A) and DiI application to M2 (B), and the photomicrograph of A merged with B (C). D, E and F: High magnification photomicrographs of FG(+) TG cells 3 days after FG application to M1 (D) and DiI application to M2 (E), and the photomicrograph of E merged with F (F). Ga: Percentage of FG and DiI double-labeled TG cells indicated by the yellow pie. White arrow heads indicate FG and DiI double-labeled TG cells, the white arrows are FG(+) TG cells and open arrow heads are DiI(+) TG cells.

We also studied the double retrograde labeling of TG neurons when M1 was labeled with FG (Fig. 6A and D) and M2 labeled with DiI (Fig. 6B and E). About 6% (5.9±1.7%, n = 7) of TG neurons labeled with FG applied into M1 were also labeled with DiI applied into M2 (Fig. 6C and G), indicating that some TG neurons innervated both M1 and M2 tooth pulps.

### 6. TRPV1 Expression in M2 TG neurons Following CFA Application to M1

**Figure 7:**
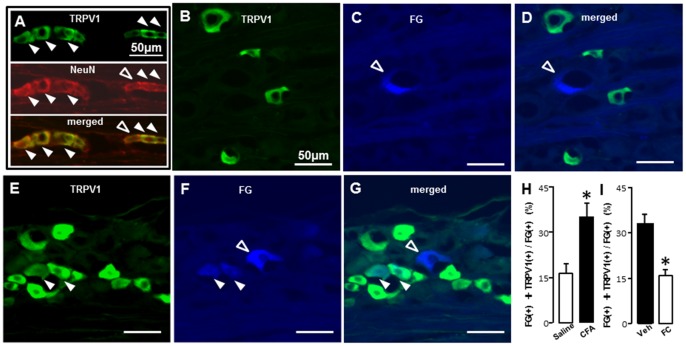
TRPV1-IR cells labeled with FG applied into M2 following CFA application to M1. A: TRPV1-IR cells (upper panel), NeuN-IR cells (middle panel) and merged photomicrographs (lower panel) in M2 capsaicin-applied rats which received CFA to M1. B-G: FG(+) TG cells expressing TRPV1-IR in M1saline-adminstrated (B, C and D) and M1 CFA-administrated (E, F and G) rats. H: The mean number of FG(+) TRPV1-IR cells/FG (+) following saline (open bur) or CFA (solid bur) application to M1. I: The mean number of FG(+) TRPV1-IR cells/FG(+) following Veh (solid bur) or FC (open bur) injection into TG in M1 CFA rats. *: p<0.05.

We studied the expression of TRPV1-IR neurons labeled with FG applying to M2 following CFA or saline application to M1, and also studied the effect of FC injection into the TG on TRPV1-IR cell expression in M1 CFA-applied rats. All TRPV1-IR cells were also showed NeuN-IR, indicating that all TRPV1-IR cells were neurons (Fig. 7A). Many FG(+) TG neurons showed TRPV1-IR in M1 CFA- and M1 saline-applied rats (saline: Fig. 7B-D, CFA: Fig. 7E-G). The mean number of FG(+) TRPV1-IR cells was significantly larger in M1 CFA-applied rats compared with M1 saline-applied rats (saline: 16.4±3.2, CFA: 35.1±4.8, n = 5 in each group) (Fig. 7 H). We observed significant decrease in the number of TRPV1-IR cells in TG following FC injection into the TG in M1 CFA-applied rats (vehicle: 28.2±3.9, FC: 15.8±2.2, n = 5 in each group) (Fig. 7 I).

## Discussion

**Figure 8:**
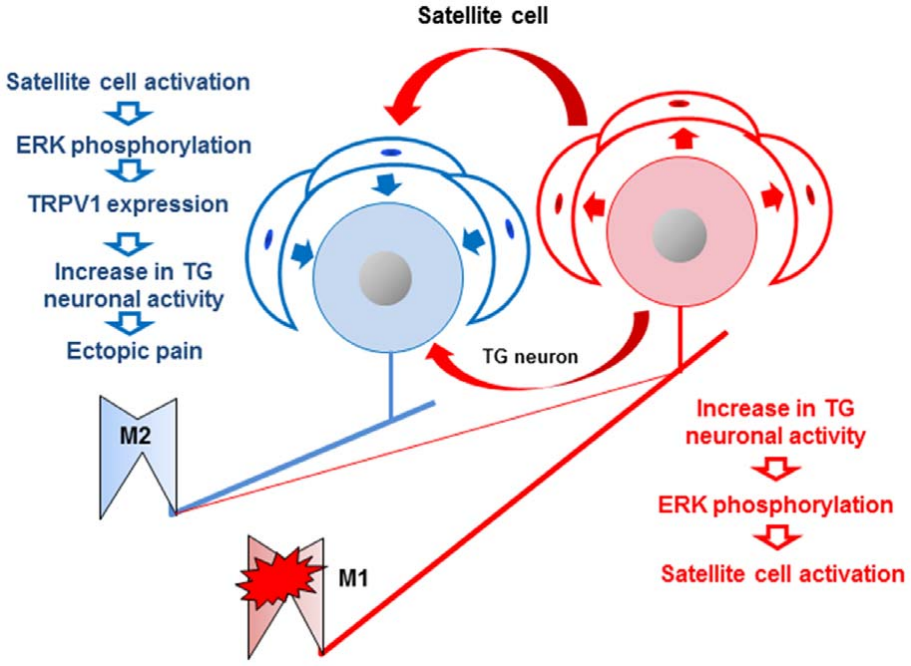
The diagram indicating proposed mechanisms of the present results. The TG cell innervating M1 is indicated by red and TG cell innervating M2 is indicated by blue. Following CFA application to M1, the excitability of TG cell innervating M2 (blue cell) is enhanced via neuron-neuron or satellite cell-neuron interaction.

We proposed the possible mechanisms based on the present findings that M1 inflammation causes the enhancement of excitability of TG neurons innervating the non-inflamed M2 as well as inflamed M1 following capsaicin application to the non-inflamed M2 tooth pulp (Fig. 8). This effect induces satellite cell activation associated with increased TRPV1-IR cells in the TG. Furthermore, some TG neurons innervating both M1 and adjacent M2 may be involved in the tooth-pulp inflammation-induced ectopic persistent pain in the tooth pulp.

### 1. Enhancement of TG neuron Excitability Following Tooth-pulp Inflammation

There was a significant increase in the number of pERK-IR TG neurons innervating the inflamed M1 compared with those innervating non-inflamed M1 following capsaicin application to M2. It has been reported that ERK is phosphorylated in DRG neurons following strong noxious stimulation of peripheral cutaneous tissues, and the number of pERK-IR DRG cells increases following increases in stimulus intensity [18]. Most of these pERK-IR cells categorized as C- or small-diameter Aδ-fibers, indicating that pERK-IR is a reliable marker of excitability of high-threshold primary afferent neurons [18]. These findings suggest that pERK-IR TG neurons innervating the M2 tooth pulp following capsaicin application to M2 become hyperexcitable in rats with M1 inflammation induced by CFA.

A barrage of action potentials is known to be generated in TG neurons following TMJ inflammation, and TG neurons innervating the inflamed TMJ become hyperexcitable with high frequency background activity [13]. A long lasting hyperexcitability of TG neurons is thought to be involved in the sensitization of TG neurons. It is also known that administration of CFA or other inflammatory agents into the TMJ capsule causes strong activation of trigeminal spinal subnucleus caudalis (Vc) nociceptive neurons, suggesting that TG neurons are activated by noxious TMJ stimuli [24], [25], [26].

The findings collectively suggest that the excitability of pERK-IR TG neurons innervating the non-inflamed M2 which received capsaicin application was strongly affected by CFA application to M1, and that peripheral activation and sensitization may occur in TG neurons innervating M2 as well as CFA-applied M1.

However, CFA and capsaicin were applied with paper points, and this procedure might cause pulpal nerve injury. Thus, the effect of pulpal nerve injury by drug application on ERK phosphorylation in TG neurons could not be rule out in this study.

### 2. Satellite Cell Hyperactivation Following Tooth-pulp Inflammation

A large number of satellite cells in TG are known to be activated following orofacial tissue inflammation or trigeminal nerve injury [27]. Swelling of the cell body and processes are typical morphological changes in the activated satellite cells [27] which release various molecules affecting the activity of TG neurons as well as resting satellite cells. In the present study, we observed many activated satellite cells in M1 CFA-applied rats compared with M1 saline-applied rats. The number of TG neurons that were encircled with activated satellite cells and that innervated the inflamed M2 or non-inflamed M2 or other oral tissues were both significantly larger in M1 CFA-applied rats compared with M1 vehicle-applied rats. Furthermore, the masseter muscle activation and ERK phosphorylation induced by M2 capsaicin application were significantly suppressed after injection of the satellite cells blocker FC into the TG. These findings suggest that satellite cells encircling TG neurons innervating both CFA-applied M1 as well as M2 are strongly activated after the M1 inflammation, resulting in the enhancement of excitability of TG neurons and the associated nocifensive behavior.

### 3. Multiple Tooth-pulp Innervations of TG neurons

Previous anatomical studies have reported that primary afferent neurons innervating the tooth pulp are somatotopically organized in the cat TG [28]. The somatotopic organization of the primary afferent neurons in TG is thought to be important in the discrimination of sensory inputs from the tooth pulp [28]. On the other hand, it has been reported that some TG neurons innervate more than 2 tooth pulps and are involved in sensory processing from multiple tooth pulps [28], [29], [30]. Such multiple tooth pulp innervations by single TG neurons may contribute to the difficulty in some clinical cases of distinguishing the tooth under pathological condition. In the present study, we observed a small number of TG neurons (6%) innervating both M1 and M2. This morphological feature of TG neurons may also be involved in the enhancement of excitability of TG neurons innervating non-inflamed tooth pulp following inflammation of adjacent teeth.

### 4. Involvement of TRPV1 Expression in Ectopic Tooth-pulp Pain

Recently, we have reported that CFA application to the lower lip causes a significant increase in TRPV1 expression in TG neurons innervating the non-inflamed whisker pad region [4]. NGF expression was enhanced in the lower-lip following CFA application to the lip, and NGF was delivered to TG neurons from the lower lip and released from TG neurons, resulting in the enhancement of TRPV1 expression in TG neurons [4]. Furthermore, satellite cell activation is also known to spread over a wide area in the TG via glia-glia interactions [17], [31]. Neuron-glia interactions are also known to be an important mechanism by which increased excitability of ganglion neurons occurs [17]. Since we observed a significant increase in the number of TRPV1-IR TG neurons innervating M2 following CFA application to M1, it is possible that the TRPV1 expression was enhanced in M2 TG neurons via satellite cell activation following CFA application to M1.

Together with previous findings, the present findings suggest that neuron-glia interactions and neuron-neuron interaction occur in the TG, and that multiple innervations by single TG neurons of tooth pulps may be involved in inflammation-induced ectopic tooth-pulp persistent pain.
